# Amyloid Plaques in Retina for Diagnosis in Alzheimer’s Patients: a Meta-Analysis

**DOI:** 10.3389/fnagi.2016.00267

**Published:** 2016-11-10

**Authors:** Jiangling Jiang, Hongyan Wang, Wei Li, Xinyi Cao, Chunbo Li

**Affiliations:** ^1^Shanghai Key Laboratory of Psychotic Disorders, Shanghai Mental Health Center, Shanghai Jiao Tong University School of MedicineShanghai, China; ^2^Bio-X Institutes, Key Laboratory for the Genetics of Developmental and Neuropsychiatric Disorders, Ministry of Education, Shanghai Jiao Tong UniversityShanghai, China

**Keywords:** Alzheimer’s disease, β-amyloid peptides, retina, diagnosis, meta-analysis

## Abstract

**Background:** Detection of retinal β-amyloid (Aβ) peptide accumulation is a novel diagnostic method for Alzheimer’s disease (AD), but there is, as yet, no conclusive evidence of its accuracy.

**Aim:** To identify the diagnostic accuracy of pathological retinal Aβ detection for AD by a meta-analytic approach.

**Methods:** Electronic and reference searches were conducted to identify studies related to the diagnostic effects of retinal Aβ detection in AD that met pre-defined inclusion criteria. The QUADAS-2 tool was employed to assess the risk of bias, and Review Manager plus the Open Meta-Analyst were used to perform the data analysis.

**Results:** From 493 unduplicated reports, five studies with small sample sizes were included in this review. Six staining methods were employed. The eligible studies showed extremely broad ranges of sensitivity (0–1.00) and specificity (0.50–1.00) with substantial heterogeneity. The estimates of positive likelihood ratio (PLR), negative likelihood ratio (NLR), diagnostic odds ratio (DOR) were also extremely varied (from 0.71 to 11.57 for PLR, from 0.04 to 1.11 for NLR, and from 0.69 to 297.00 for DOR).

**Conclusions:** The limited number of eligible studies and their methodological heterogeneity make it impossible to come to a conclusion whether pathological retinal Aβ detection is an effective diagnostic tool for AD. More studies, especially large surveys investigating retina Aβ load with quantitative methods among consecutive or random samples, are needed to determine the accuracy of Aβ detection for diagnosing AD.

## Introduction

Alzheimer’s disease (AD) is a progressive brain disorder that damages brain cells, which leads to memory loss and other brain dysfunctions (Kandimalla et al., 2013; Ramesh et al., 2014; Jansen et al., 2015). Globally, AD is the most frequent neurodegenerative disorder and accounts for 50–70% cases of dementia (Winblad et al., 2016). It was estimated that dementia affected 46.8 million individuals and cost 818 billion USD worldwide in 2015 (Alzheimer’s Association, 2015).

At present, the definitive diagnosis of AD still depends on an autopsy of the brain, by the histopathological identification of amyloid precursor protein’s (APP) hallmark proteolytic products, β-amyloid (Aβ) peptides, and intracellular neurofibrillary tangles (Sisodia and Price, 1995; Hardy and Selkoe, 2002). According to the Aβcascade hypothesis, the principal event in the pathogenesis of AD is the accumulation of Aβ plaques in the brain (Hardy and Selkoe, 2002; Jack et al., 2010; Karran et al., 2011). Thus, massive attention has been paid to the detection of Aβ accumulation among common AD biomarkers.

As an extension of the central nervous system, the retina is easily accessed through widely used imaging techniques such as scanning laser ophthalmoscopy (SLO) and optical coherence tomography (OCT). Therefore the retina is likely to be an ideal target for non-invasive imaging of AD *in vivo*, provided that Aβ plaques accumulate in the retinas of AD patients and that their properties are consistent with those in the brain (Koronyo et al., 2012). As a consequence, recent studies have focused on identifying Aβ in retina as a technique to facilitate the diagnosis of AD in humans and in animal models (Koronyo-Hamaoui et al., 2011; Koronyo et al., 2012).

Koronyo-Hamaoui et al. (2011) stated that they had demonstrated Aβ accumulation in postmortem retinas from AD patients, while Schön et al. (2012) could not detect any Aβ plaques in the retinas of AD patients. Obviously, the interaction between retina Aβ and brain Aβ has not been articulately illuminated. Hence, the importance of the postmortem tests for Aβ in the retina would be to guide future research concerning non-invasive retinal Aβ detection techniques. In this meta-analysis, we aimed to determine the accuracy of pathological Aβ detection for diagnosing AD.

## Materials and Methods

### Search Strategy

We searched BIOSIS Previews (ISI Web of Knowledge), Current Contents Connect (ISI Web of Knowledge), EMBASE(Ovid SP), MEDLINE (Ovid SP), Science Citation Index (ISI Web of Knowledge), and PsycINFO (Ovid SP) up to March 16, 2016. We considered using Chinese-language databases (CNKI, CQVIP, Wanfang), however, we decided not to use these databases in our formal search since no relevant reports were identified among them in the preliminary searches. A structured search strategy was devised for each platform using following key words and their abbreviation and MeSH synonyms: (1) Alzheimer’s disease; (2) β-amyloid; (3) retina; (4) pathologic or histologic or immune or fluorescent test. Detailed electronic search strategies were presented in Supplementary Table S1. For reference lists, all eligible published reports were scanned for further possible titles. This procedure was repeated until no new titles were found (Greenhalgh and Peacock, 2005; Horsley et al., 2011).

### Selection of Studies

Two review authors (JJ, HW) independently performed assessments of titles and abstracts to identify potentially eligible studies for full-text reviews. They then performed further assessment of full manuscripts against the inclusion criteria, which were as follows: (1) the target condition is AD, which should be confirmed by the neuropathological tests of brain tissue, and neuropathological information based on the Braak (Braak et al., 2006), the Consortium to Establish a Registry for Alzheimer’s Disease (CERAD) (Mirra et al., 1991), or the National Institute for Aging and the Ronald and Nancy Reagan Institute for the Alzheimer’s Association (NIA-RIA) criteria (Ball et al., 1997), which are all recognized as acceptable confirmations of AD dementia (Murayama and Saito, 2004); (2) the index test in this review is the presence of Aβin the retinas, assessed by any kinds of routine staining; (3) we only considered cross-sectional studies because the index test is usually conducted posthumously due to its invasive nature. When necessary, a third review author (WL) acted as an arbitrator to resolve disagreements that could not be resolved through discussion by the original two reviewers. When the same data set was presented in two or more papers, the primary paper with the largest number of patients or the most informative data, was included. At each time point of this selection process, the numbers of studies selected were detailed in a Preferred Reporting Items for Systematic reviews and Meta-Analyses (PRISMA) flow diagram (Moher et al., 2009).

### Data Extraction and Quality Assessment

The two review authors independently extracted the data on study characteristics, including the following information: bibliographic details of the primary paper, patient-sampling details, basic patient characteristics, details of the index test, target conditions, reference standards, and data for the 2 × 2 tables. Again, a third review author acted as an arbitrator to settle disagreements when necessary.

The investigators identified the methodological quality of each study using QUADAS-2 (Whiting et al., 2011). Instead of applying QUADAS-2 data to forming a summary quality score, a narrative summary was generated that included studies that found a high/low/unclear risk of bias and concerns with regard to applicability. We refined the original QUADAS-2 tools to meet the needs of this systematic review. Since the index test in this review is qualitative, the item ‘If a threshold was used, was it pre-specified?’ in the index test domain was recognized as not applicable. Additionally, the question ‘Was there an appropriate interval between index test and reference standard?’ in the flow and timing domain was not used because we only included cross-sectional studies.

### Statistical Analysis

The data of the 2 × 2 tables for the index test performance (True positive, false negative, false positive, true negative) were employed to calculate the accuracy estimates of each primary study. By utilizing Review Manager version 5.3, we calculated each data set’s sensitivity (Sen), specificity (Spe), and 95% confidence interval (95% CI). Using Open Meta-Analyst build 5.26.14, we estimated each study’s positive likelihood ratio (PLR), negative likelihood ratio (NLR), diagnostic odds ratio (DOR), and 95% CI, respectively, with a random effect approach. The estimate results were presented graphically in a forest plot. Additionally, the sensitivity and specificity estimates with their 95% CI among those studies were also presented in a receiver operating characteristic (ROC) space. We planned not to compute or plot the pooled point estimates of Sen and Spe with the hierarchical summary ROC curve (HSROC) method (Rutter and Gatsonis, 2001) or the bivariate random effects approach (Reitsma et al., 2005), since threshold effects were usually not identified in a qualitative index test. Sensitivity analyses were performed with or without inclusion of possible AD and probable AD.

### Heterogeneity Investigation and Reporting Bias Assessment

The potential sources of heterogeneity include patient factors, differing assay methods for the index test, variety in reference standards, how the primary studies operated, and so forth. All of these factors may affect the diagnostic accuracy of the test. Statistical heterogeneity was assessed via visual inspection of the forest plot and the ROC plot and by utilizing *I*^2^ alongside the χ^2^ *P*-value. If *I*^2^ was greater than or equal to 50% with a statistically significant χ^2^ result, we considered the data to have substantial levels of heterogeneity (Higgins and Green, 2008). When significant heterogeneity was identified, we planned to investigate the reasons for heterogeneity by the meta-regression approach and visual inspection. Due to current uncertainty about how reporting-bias operates in test accuracy studies, we did not investigate it by interpretation of funnel plots or other existing analysis tools.

## Results

### Eligible Studies

**FIGURE 1:**
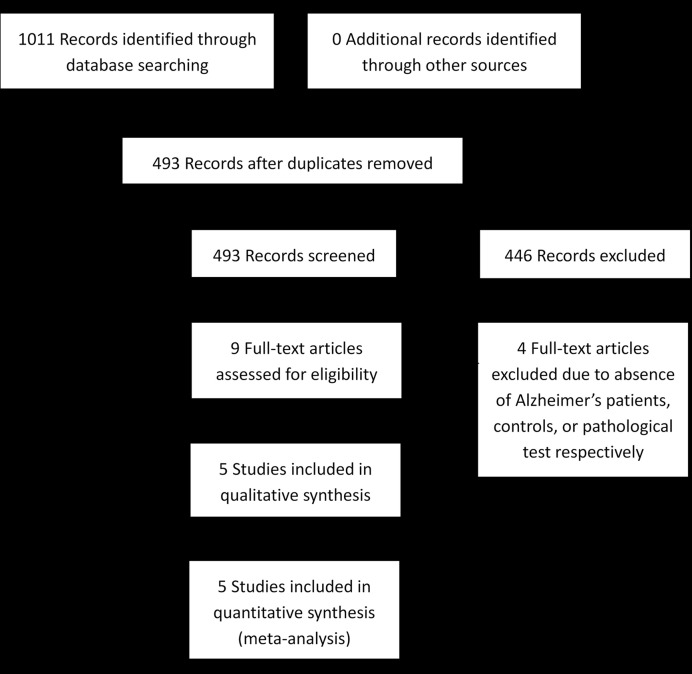
**Flow chart of study selection**.

From 1,011 records identified via electronic searching, only five eligible studies were eventually included (Koronyo-Hamaoui et al., 2011; Schön et al., 2012; Ho et al., 2014; Tsai et al., 2014; La Morgia et al., 2016). The literature selection process is detailed in **Figure 1**. Among five included studies, six staining methods were employed, including Congo red along, Aβ antibody alone, Aβ antibody plus curcumin, Aβ antibody plus FSB [(*trans*, *trans*)-1-fluoro-2,5-bis(3-hydroxycarbonyl-4-hydroxy)styrylbenzene], Aβ antibody plus Thioflavin-S, and Aβ antibody plus Thioflavin-T. Six anti-Aβ clones were used, with three of them against the mid-portion of Aβ, two against C-terminus, and one against N-terminus. Only three studies differentiated definite AD patients from possible or probable AD patients. The median age in Schön’s research (56 years old) was substantially lower than in other eligible literature (≥71 years old). The characteristics of the included studies are presented in **Table 1**. It should be noted that samples of different staining methods from the same study were usually identical. Whole mount retina was used in Koronyo-Hamaoui’s and Tsai’s while other three researches used cross-section tissue.

**Table 1:** Characteristics of included studies.

Study	Reference standard	Staining method	Anti- Aβ clone (targeted amino acid residues)	dAD only	Number of subjects	Median age	Gender (female:male)
Koronyo-Hamaoui et al., 2011	B, C, N	Abs, Abs+C Abs+T-S	6E10 (1–16) DE2B4 (1–16) 4G8 (17–24) 11A5B10 (34–40) 12F4 (36–42)	Yes No	13 18	79.0 79.0	4:9 7:11
Schön et al., 2012	B, C	Abs Abs+FSB Abs+T-S	4G8 (17–24)	No	10	56.5	3:7
Ho et al., 2014	B, C	CR	6F3D (8–17)	Yes	15	79.0	11:4
		Abs		No	17	82.0	12:5
Tsai et al., 2014	B, C, N	Abs	6E10 (1–16)	No	12	80.0	6:6
		Abs+T-T			4	79.0	-
La Morgia et al., 2016	B, C, N	Abs	6E10 (1–16)	Yes	10	73.8	5:5

*Abs, C, CR, FSB, T-S, T-T in index test column refer to antibodies, curcumin, Congo red, (trans, trans)-1-fluoro-2,5-bis(3-hydroxycarbonyl-4-hydroxy)styrylbenzene, Thioflavin-S, and Thioflavin-T, respectively; B, C, N from reference standard refer to the Braak, the Consortium to Establish a Registry for Alzheimer’s Disease (CERAD), and the National Institute for Aging and Ronald and Nancy Reagan Institute for the Alzheimer’s Association (NIA-RIA) criteria, respectively; dAD, is the abbreviations of definite Alzheimer’s disease.*

The results of quality assessment are summarized in **Table 2**. All of the eligible studies were labeled low applicability concerns in every domain. However, they all shared the same methodological limitations: insufficient information about the exclusion criteria and whether blinding assessment was employed, and case-control designs used without stating whether the research recruited consecutive or random samples.

**Table 2:** Results of quality evaluation.

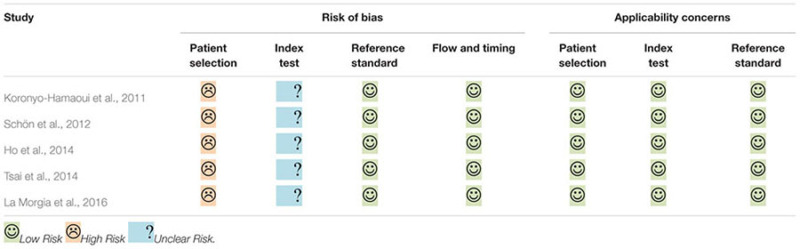

### Results of Data Analysis

We classified the staining methods as immunolabeling and fluorescent staining (with specific anti-Aβ compounds), and then estimated Sen, Spe, PLR, NLR, DOR, and 95% CI by each method category. We excluded Congo red staining data from the analysis because it is not a specific compound of Aβ. If a study had two or more data sets classified into a single method, then the medians instead of the original data were employed for analysis.

**FIGURE 2:**
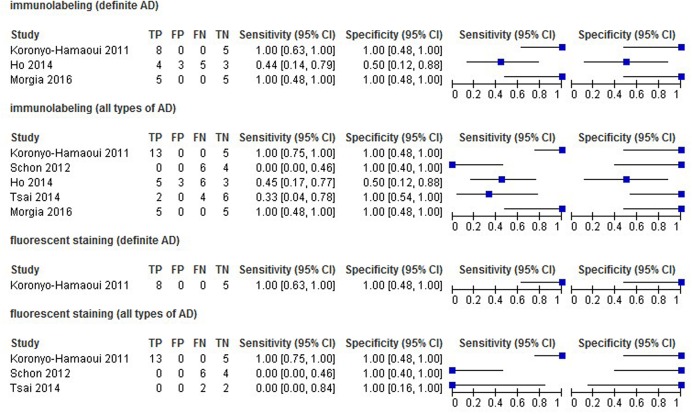
**Forest plot of data analysis results of sensitivity and specificity**.

The estimates of Sen and Spe were extremely varied (from 0.00 to 1.00 for Sen, and 0.50 to 1.00 for Spe) with a wide 95% CI. The estimate results were detailed in a forest plot (**Figure 2**) and a ROC plot (**Supplementary Figure S4**). If more than one study applied a certain kind of staining method (in this review, immunostaining with and without the inclusion of probable or possible AD patients, and fluorescent staining for all types of AD patients), we included those studies in the data synthesis. However, due to substantial heterogeneity and the small number of included studies, the data synthesis results are presented in **Supplementary Figures S1**–**S3** instead, and were not taken into account when drawing the conclusions.

**FIGURE 3:**
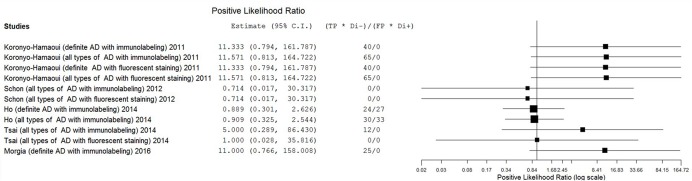
**Forest plot of data analysis results of positive likelihood ratio**.

**FIGURE 4:**
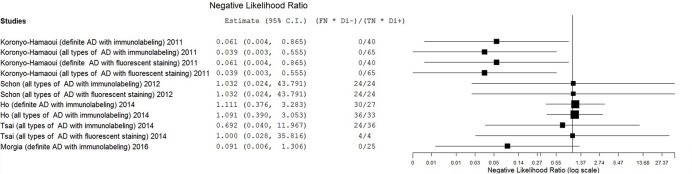
**Forest plot of data analysis results of negative likelihood ratio**.

**FIGURE 5:**
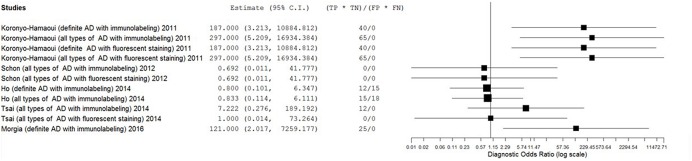
**Forest plot of data analysis results of diagnostic ratio**.

The estimates of PLR, NLR, and DOR were also varied extremely (from 0.71 to 11.57 for PLR, from 0.04 to 1.11 for NLR, from 0.69 to 297.00 for DOR) with wide 95% CI. The estimate results were detailed in a forest plot (**Figures 3**–**5**). Data synthesis of these 3 measures failed because there were too many zeroes in the data sets and the number of available studies was too small.

### Results of Heterogeneity Investigation

The meta-regression approach, with target population (definite AD only or all types of AD), tissue preparation (whole mount or cross section), staining methods (immunolabeling or fluorescent staining), sex ratio (female:male), median age as an independent variable and DOR as dependent variable, was employed to investigate the sources of heterogeneity using a random model.

For target population, meta-regression was conducted only using paired data sets from studies including both kinds of target population (Koronyo-Hamaoui’s and Ho’s) to reduce the influence of between-study confounding factors. Only those data sets using immunolabeling were included, in order to exclude the impact of different staining methods, and fluorescent staining data were not used since Ho’s study did not employ this method. The coefficient was 0.20 with a p-value of 0.943.

For the staining methods, meta-regression was performed among paired data sets from studies utilizing both immunostaining and fluorescent staining (Koronyo-Hamaoui’s, Schön’s, and Tsai’s). The coefficient was 0.39 with a *p*-value of 0.84.

For tissue preparation, gender ratio, and median age, we only included data sets using immunolabeling for all types of AD patients, in order to reduce the influence of staining methods or other confounding factors. These categories of data sets were selected because they contained the largest sample sizes. Logarithmic transformation was applied before the analysis of gender ratio data. The estimates of coefficients were -2.78, 1.11, and -0.04, and the estimates of *p*-values were 0.09, 0.65, and 0.75 for tissue preparation, gender ratio, and median age, respectively.

By visual inspection of the DOR forest plot, we found that for a certain study using a certain kind of AD population, different staining methods did not change the estimate results with only one exception (Tsai’s).

## Discussion

### Main Findings

The primary goal of this meta-analysis review was to determine the ability of pathological detection of Aβin the retina to diagnose AD. Although some studies have recently shown promising outcomes with ocular Aβtests for AD diagnosis (Frost et al., 2014; Kerbage et al., 2014), the results were relatively disappointing in this review. The most promising results came from Koronyo-Hamaoui’s study (Sen = 1.00, Spe = 1.00, NLR ≤ 0.06, PLR ≥ 11.33, DOR ≥ 187.00) and La Morgia’s study (Sen = 1.00, Spe = 1.00, NLR = 0.09, PLR = 11.00, DOR = 121.00). Point estimates of the data set from Tsai’s research using immunolabeling in all types of AD population were less promising (Sen = 0.33, Spe = 1.00, NLR = 0.69, PLR = 5.00, DOR = 7.22). Moreover, results from other data sets showed that the accuracy of retinal Aβ detection was very poor (Sen ≤ 0.45, NLR ≥ 1.00, PLR ≤ 1.00, DOR ≤ 1.00). This inconsistency between studies and the significant statistical heterogeneity indicated that there was still no conclusion to be drawn about whether retinal Aβ detection is a suitable tool for the diagnosis of AD.

Additionally, we aimed to investigate heterogeneity and its sources. The quality assessment provided few clues about heterogeneity source. The qualitative nature of the pathologic tests did not support the hypothesis that threshold effects were the main cause of the heterogeneity. Results of both visual inspection and meta-regression did not support the idea that staining method differences were a main source of heterogeneity. The meta-regression approach provided little evidence except for a trend in tissue preparation and such trend was not supported by the result of visual inspection. It must be noticed that Koronyo-Hamaoui used five antibody clones (against the N-terminus, the mid-portion, or the C-terminus of the amino acid) to label Aβ, while other four studies used only one clone (against either the N-terminus or the mid-portion). The difference in anti-Aβ is likely to contribute to the heterogeneity. Unfortunately, the limited number of studies hampered us from confirming this hypothesis and figuring out if the cause of heterogeneity lay in the number of clones, the targeted locus, or other attributes of these clones.

### Limitations

Several factors limited this systematic review. The most notable one was the small quantity of available studies, which had a tremendous impact on the methods selected for pooled estimates and on the heterogeneity investigation, as well as an enormous impact on the preciseness and reliability of our results. Another limitation and a possible reason for the small number of eligible studies was restricting the literature search to the English and Chinese language. Although there is no sufficient evidence of a systematic bias induced by language restrictions for meta-analysis, and the quality of English-language studies may exceed that of other languages, the inclusion of non-English research would likely improve the precision of the outcome estimates (Morrison et al., 2012). Third, subgroup analyses were not utilized to explore sources of heterogeneity. And we failed to investigate other potential sources, such as fixation chemistry and application of antigen retrieval. Again, these limitations were the consequence of the lack of eligible studies. Hence, it was impossible to reach a satisfactory explanatory power via our investigation of heterogeneity sources. Fourth, all eligible study reported qualitative data instead of quantitative Aβ load that could reveal more useful information.

### Implications

This meta-analytic study suggested that for now evidences are not sufficient to conclude whether pathological retinal Aβ detection is an effective diagnostic tool for AD. Hinton reported a lack of Aβ deposition in postmortem retinas of AD patients in 1986 (Hinton et al., 1986). Nevertheless, only a few studies have further explored the relationship between AD and Aβ in the retina by pathologic tests since then, while a great deal of effort has been made to develop *in vivo* retinal Aβ detectors for the diagnosis of AD. It is possible that the eager demand for novel non-invasive diagnostic tools and the preference for positive results led to this disparity that transgenic mouse models and *in vivo* human research showed Aβ aggregation in the retina (Dutescu et al., 2009; Perez et al., 2009; Alexandrov et al., 2011; Frost et al., 2014) but only mixed results was found in postmortem human research. The lack of postmortem retinal samples from AD patients may also contribute to this inconsistency.

We hypothesized that, as in other fields of disease diagnosis, pathological tests would be the golden standard for the detection of Aβ, either in the brain or the retina, despite the fact that *in/ex vivo* differences might affect the test results. Since autopsies do not show considerable accuracy, for now, it may be worth reconsidering the development of *in vivo* detection methods for retinal Aβ. For instance, the differences in pathological changes between AD patients and animal models may result in disparities between clinical assessments and animal experiments, and the ligands could be bound to other types of receptors that could be falsely recognized as Aβ. A strong correlation between AD and retina Aβ load must be demonstrated before we could consider retina Aβ as a validated biomarker of AD and develop reliable diagnostic tools base on retina Aβ detection.

We were unable to identify the main contributor to the sharp discrepancy of results between studies. The variance in research methodology, including sample characteristics and labeling methods, was substantial and may strongly affect the results. Based on very limited evidences, whole mount retinas labeled by various antibodies that target both the terminus and the mid-portion of Aβ seems to be the most promising detection method. However, this is not conclusive and yet needs to be tested. Thus the potential confounding factors mentioned above, embedding methods, length of disease, or other possible factors that were not considered in this study should be further investigated in future research. Quantitative methods rather than qualitative ones would be preferred. Consecutive or random sample recruitment should be specified, blinding of results between conductors of index tests and reference tests should be employed if possible, and any exclusion criteria should be described in detail.

## Author Contributions

CL designed the study. JJ, HW, and WL collected the data. JJ performed all analyses. JJ, HW, WL, and XC wrote the manuscript. All authors contributed to writing of this manuscript.

## Conflict of Interest Statement

The authors declare that the research was conducted in the absence of any commercial or financial relationships that could be construed as a potential conflict of interest.

## Supplementary material

FIGURE S1 **Forest plot of data synthesis results of immunostaining for definite AD patients**.

FIGURE S2 **Forest plot of data synthesis results of immunostaining for all types of AD patients**.

FIGURE S3 **Forest plot of data synthesis results of immunofluorescence for all types of AD patients**.

FIGURE S4 **ROC plot of data analysis results**.
