# Supplementary figures and images for: Amyloid Plaques in Retina for Diagnosis in Alzheimer’s Patients: a Meta-Analysis
Source: Front Aging Neurosci. 2016 Nov 10;8:267. doi: 10.3389/fnagi.2016.00267 (PMC5102884; doi:10.3389/fnagi.2016.00267)

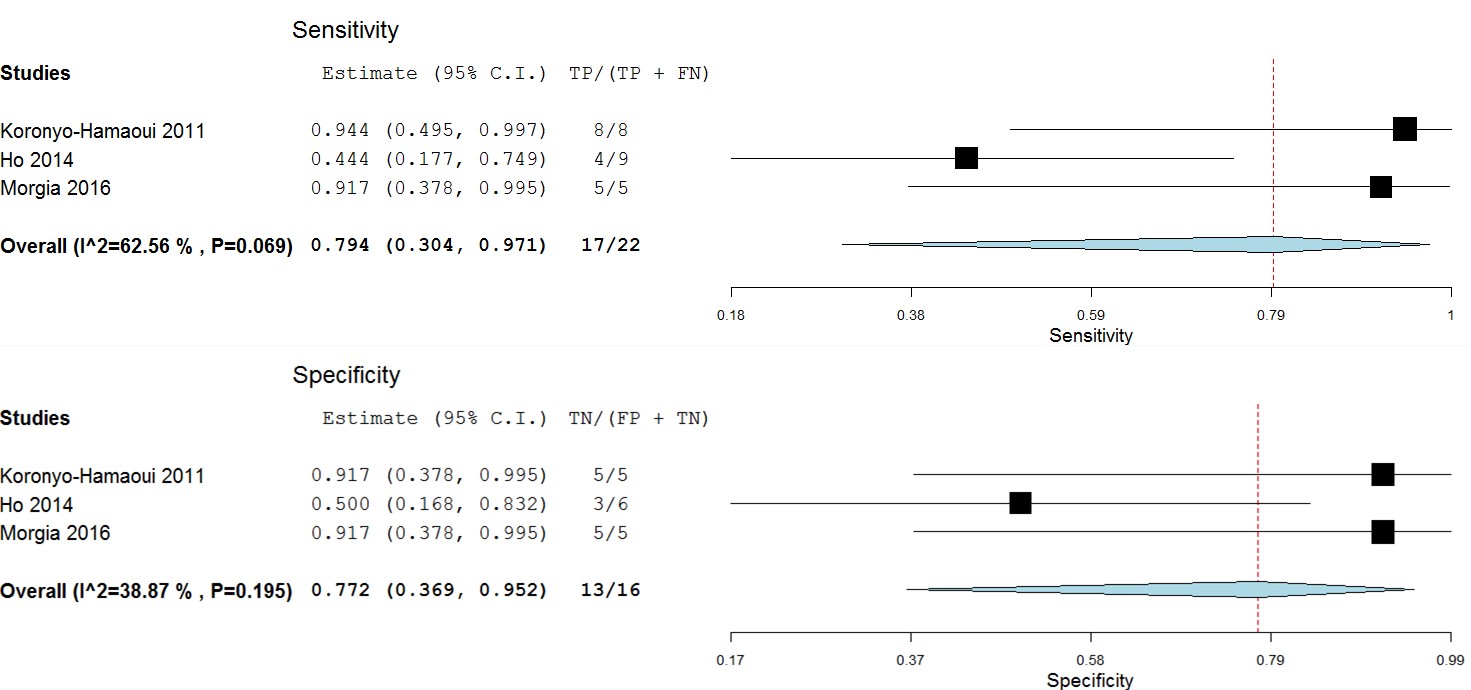

Supplement: FIGURE S1 — Forest plot of data synthesis results of immunostaining for definite AD patients. [file Image_1.JPEG]

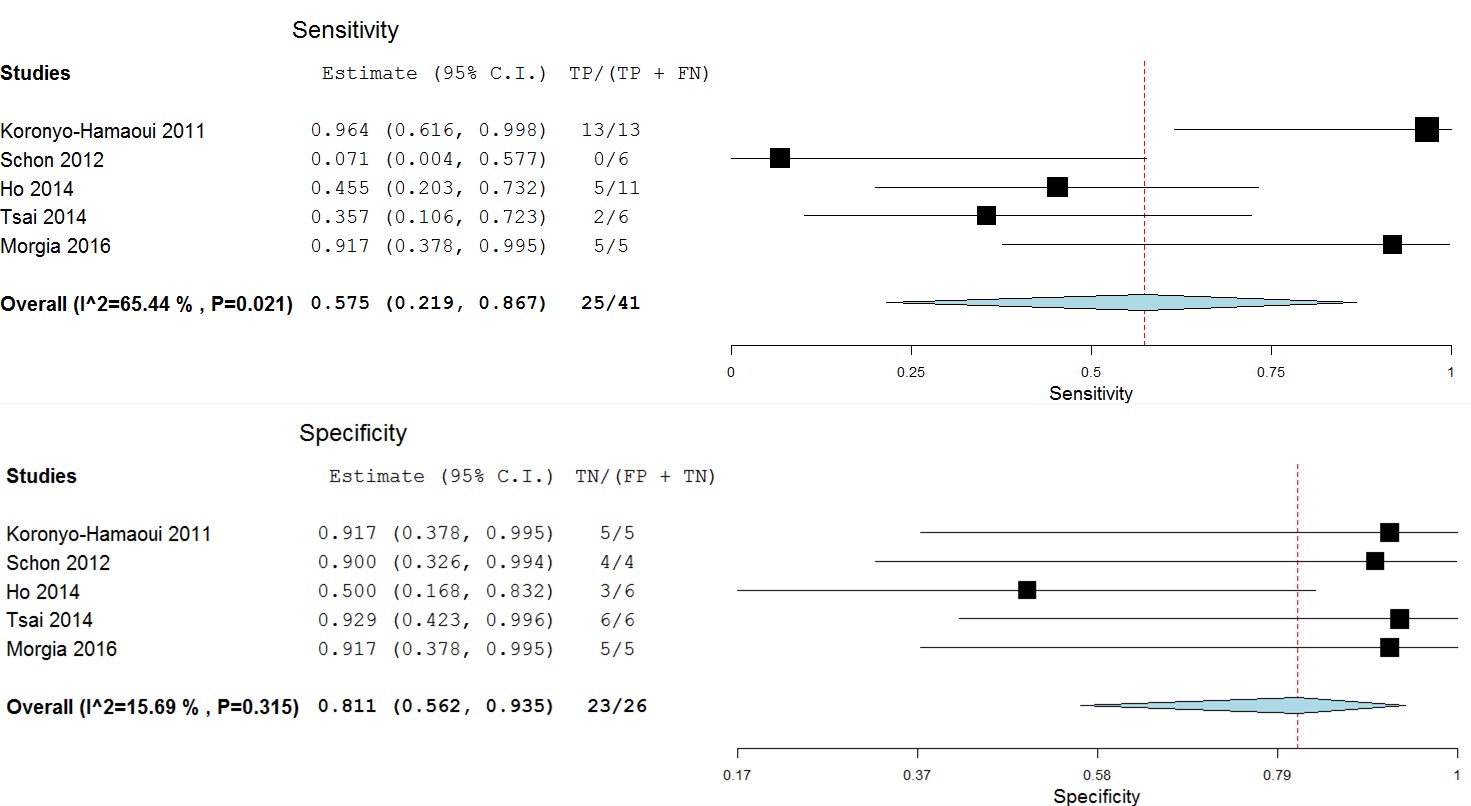

Supplement: FIGURE S2 — Forest plot of data synthesis results of immunostaining for all types of AD patients. [file Image_2.JPEG]

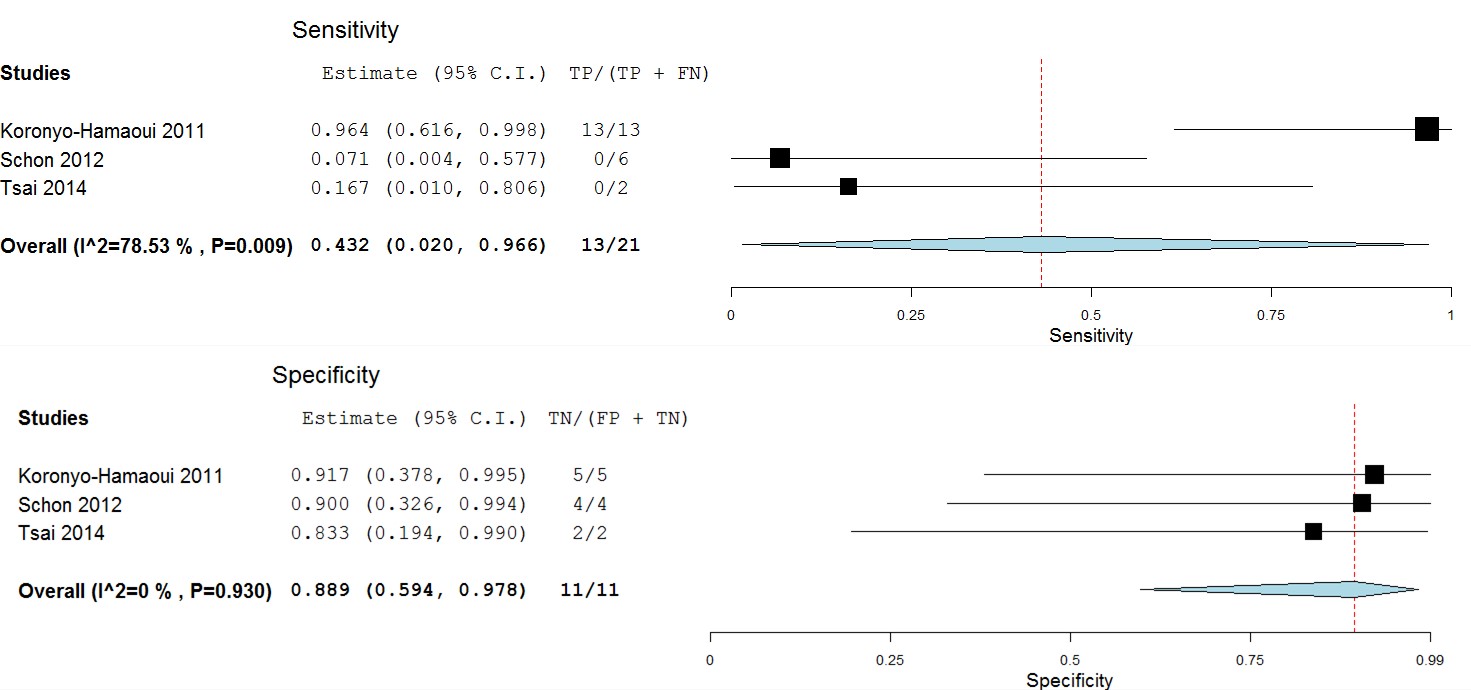

Supplement: FIGURE S3 — Forest plot of data synthesis results of immunofluorescence for all types of AD patients. [file Image_3.JPEG]

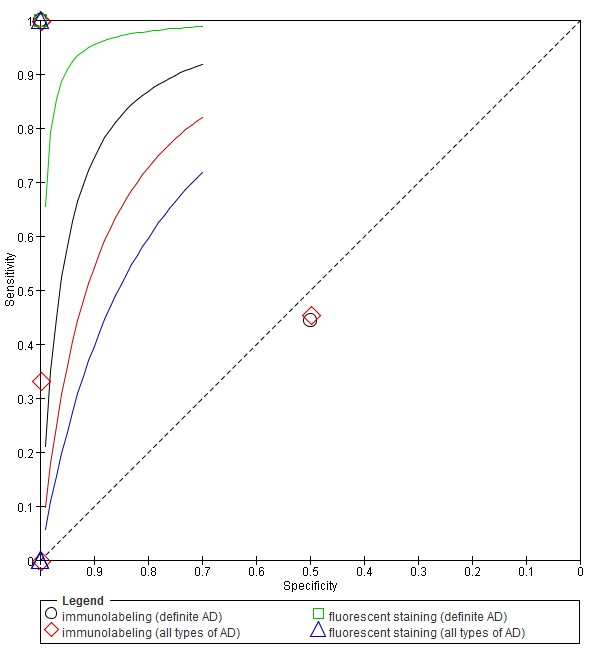

Supplement: FIGURE S4 — ROC plot of data analysis results. [file Image_4.JPEG]
