# Supplementary material for: Amyloid Plaques in Retina for Diagnosis in Alzheimer’s Patients: a Meta-Analysis
Source: Front Aging Neurosci. 2016 Nov 10;8:267. doi: 10.3389/fnagi.2016.00267 (PMC5102884; doi:10.3389/fnagi.2016.00267)
Supplement: Supplementary file 5 [file Table_1.DOCX]

| Key words | Ovid SP | ISI Web of Knowledge |
| --- | --- | --- |
| Alzheimer’s disease | (Alzheimer or ((Presenile or Senile) adj2 Dementia)).ti,ab. | TS=(Alzheimer or ((Presenile or Senile) near/2 Dementia)) |
| β-amyloid | ((Alzheimer adj2 beta) or (Alzheimer adj2 amyloid) or (beta adj2 amyloid) or ((Alzheimer or beta or amyloid) adj2 plaque) or "ABP" or "AD AP" or "Aβ" or "β amyloid" or "abeta" or "a beta").ti,ab. | TS=((Alzheimer near/2 beta) or (Alzheimer near/2 amyloid) or (beta near/2 amyloid) or ((Alzheimer or beta or amyloid) near/2 plaque) or "ABP" or "AD AP" or "Aβ" or "β amyloid" or "abeta" or "a beta") |
| retina | (retina or retinal or "Fundus Oculi" or "Macula Lutea" or "Fovea Centralis" or "Optic Disk").ti,ab. | TS=(retina or retinal or "Fundus Oculi" or "Macula Lutea" or "Fovea Centralis" or "Optic Disk") |
| pathologic or histologic or immune or fluorescent test | neuropahologic* or pathologic* or histologic* or histopathologic* or immun* or anti* or fluor?metric or fluorescen* | TS=(neuropahologic* or pathologic* or histologic* or histopathologic* or immun* or anti* or fluor?metric or fluorescen*) |

**Supplementary table 1. Detailed electronic search strategies**
